# The role of the circle of Willis in internal carotid artery stenosis and anatomical variations: a computational study based on a patient-specific three-dimensional model

**DOI:** 10.1186/s12938-015-0105-6

**Published:** 2015-11-25

**Authors:** Guangyu Zhu, Qi Yuan, Jian Yang, Joon Hock Yeo

**Affiliations:** School of Energy and Power Engineering, Xi’an Jiaotong University, 28 Xian Ning West Rd, Xi’an, 710049 Shaanxi China; Department of Radiology and Medical Imaging, The First Affiliated Hospital of Xi’an Jiaotong University, 277 Yanta West Road, Xi’an, 710061 Shaanxi China; School of Mechanical and Aerospace Engineering, Nanyang Technological University, 50 Nanyang Ave, Singapore, 639798 Singapore

**Keywords:** Circle of Willis, Cerebral blood supply, Collateral mechanism, Anatomical variations

## Abstract

**Background:**

The aim of this study is to provide better insights into the cerebral perfusion patterns and collateral mechanism of the circle of Willis (CoW) under anatomical and pathological variations.

**Methods:**

In the current study, a patient-specific three-dimensional computational model of the CoW was reconstructed based on the computed tomography (CT) images. The Carreau model was applied to simulate the non-Newtonian property of blood. Flow distributions in five common anatomical variations coexisting with different degrees of stenosis in the right internal carotid artery (RICA) were investigated to obtain detailed flow information.

**Results:**

With the development of stenosis in unilateral internal carotid artery (ICA), the cerebral blood supply decreased when the degree of stenosis increased. The blood supply of the ipsilateral middle cerebral artery (MCA) was most affected by the stenosis of ICA. The anterior communicating artery (ACoA) and ipsilateral posterior communicating artery (PCoA) functioned as the important collateral circulation channels when unilateral stenosis occurred. The blood flow of the anterior circulation and the total cerebral blood flow (CBF) reached to the minimum in the configuration of the contralateral proximal anterior cerebral artery (A1) absence coexisting with unilateral ICA stenosis.

**Conclusions:**

Communicating arteries provided important collateral channels in the complete CoW when stenosis in unilateral ICA occurred. The cross-flow in the ACoA is a sensitive indicator of the morphological change of the ICA. The collateral function of the PCoA on the affected side will not be fully activated until a severe stenosis occurred in unilateral ICA. The absence of unilateral A1 coexisting with the stenosis in the contralateral ICA could be the most dangerous configuration in terms of the total cerebral blood supply. The findings of this study would enhance the understanding of the collateral mechanism of the CoW under different anatomical variations.

## Background

Brain ischemic infarction occurs when the cerebral perfusion reduced below a certain threshold, such as in the situation of a sudden occlusion of the feeding artery, a rupture of intracranial aneurysms, the embolic phenomena or the surgical maneuvers. Cerebral perfusion depends not only on the status of the affected vessels but also the collateral capacity of collateral pathways [1]. The circle of Willis (CoW), a ring-like arterial structure located at the base of the brain, is a cerebral blood supply path [2] as well as a primary cerebral collateral flow channel. It consists of a single anterior communicating artery (ACoA), paired anterior cerebral arteries (ACA), middle cerebral arteries (MCA), internal carotid arteries (ICA), posterior communicating arteries (PCoA), posterior cerebral arteries (PCA), single basilar artery (BA) and vertebral arteries (VA).

Based on the clinical observations, however, congenital incompleteness of the CoW was observed in over 50 % of the population [3–5]. Typical anatomical variations include the absence of ACoA, unilateral or bilateral PCoA, fetal type arteries, and fused vessels [6]. Such variations undermine the compensational capability of the cerebral arteries and subsequently result in undesirable clinical consequences, including transient ischemic attack (TIA) and ischemic stroke. When the variations coexist with surgical clamping or ICA stenosis, the risks are even higher. Therefore, a detailed knowledge of the cerebral blood distribution and collateral flow patterns of the CoW under anatomical and pathological variations is important for understanding the collateral mechanism of the CoW, and also valuable for early diagnosis and pre-operation planning of the cerebral vascular diseases.

Clinically, the use of the transcranial doppler (TCD) [7–18], magnetic resonance angiography (MRA) [11, 13, 15, 17, 19–24] and computed tomography angiography (CTA) [20, 25–27] provided an in vivo perspective of the role of the CoW in the collateral flow under anatomical variations. Numerically, simulations based on complicated mathematical approaches have long been used in previous works [28–30] as well as recent studies [31–35] in order to obtain more detailed flow patterns in the CoW. A number of numerical models have been developed for this purpose, including one-dimensional models [36–38], two-dimensional models [2, 39], three-dimensional models [37, 40–42] and patient-specific models [43]. These clinical and numerical studies provided good insights into the blood flow in the CoW under a variety of anatomical and pathological variations. However, the studies aimed to investigate the impact of growing plaque in the ICA on the cerebral perfusion were limited. Also, there is a lack of information about the collateral mechanism under such conditions.

In this paper, a three-dimensional patient-specific model of the CoW was reconstructed from clinical CT images. Based on the model, the study was carried out to assess the cerebral perfusion patterns and to explore the collateral mechanism of the CoW under anatomical variations coexisting with growing stenosis in the unilateral ICA. The validation of the current work was conducted according to the CFD validation guide [44].

## Methods

### Model preparation

The entire head of the healthy volunteer was scanned with a 64-detector row spiral CT scanner (Aquilion 64, Toshiba Medical Systems, CA, USA) in the first Affiliated Hospital of Xi’an Jiaotong University (Xi’an, China). Multidetector CT (MDCT) angiographies were obtained with the following parameters: 0.5 mm slice thickness, 120 kV tube voltage, and 350 mA tube current. The CT images were stored as standard DICOM format.

**Fig. 1:**
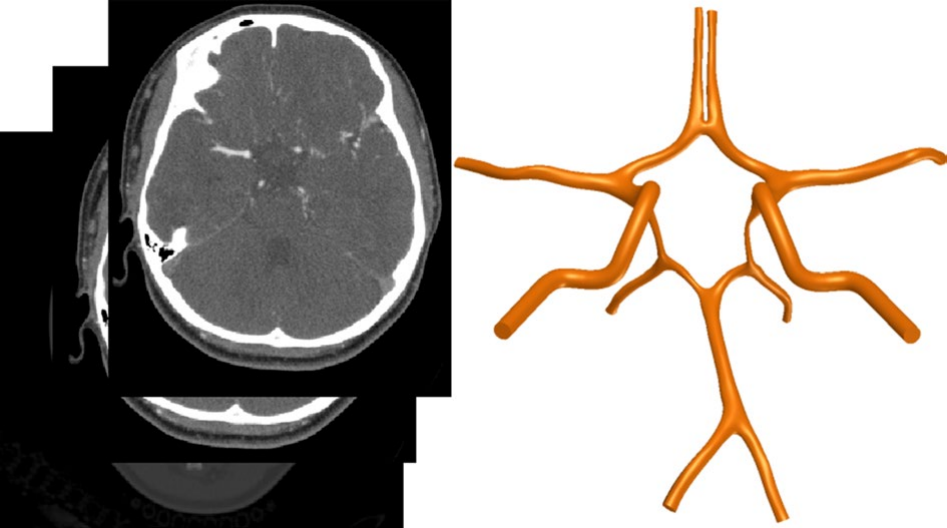
Digital model of the circle of Willis

The segmentation and reconstruction of the CTA images were conducted with the aid of commercial software MIMICS (Materialise Inc., Leuven, Belgium). After the arteries were separated from the surrounding bone and soft tissues, a complete digital model of the CoW was constructed and exported as STL format (Fig. 1).

**Fig. 2:**
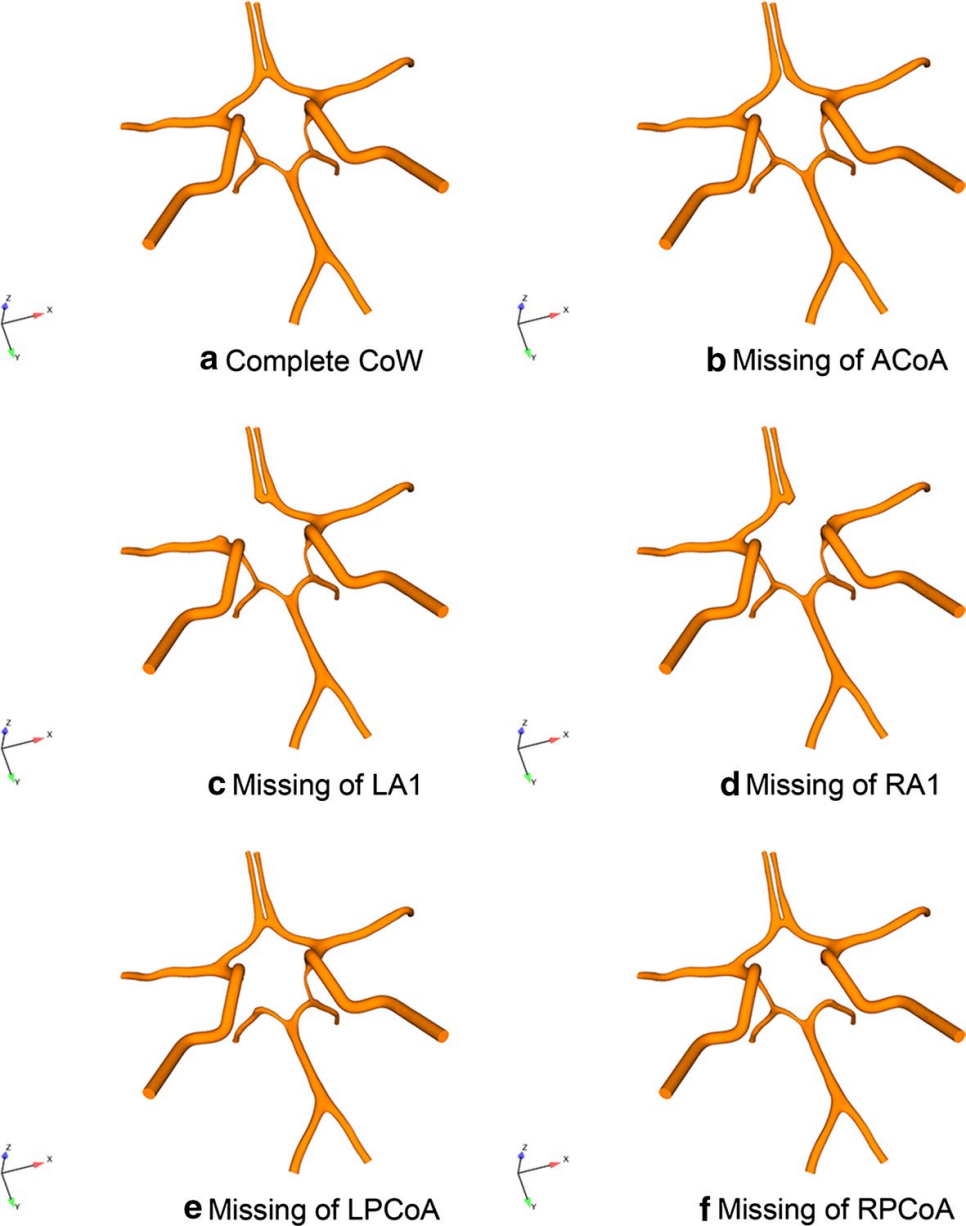
Anatomical variations studied in present study

Based on the complete patient-specific model, frequent anatomical variations were prepared. The variations considered in the current study include the absence of the ACoA, right ACA-A1 (RA1), left ACA-A1 (LA1), right PCoA (RPCoA) and left PCoA (LPCoA), respectively (Fig. 2).

Coexisting with the variations, six different degrees of stenosis, namely 0 % (no stenosis), 25, 50, 75, 90 and 100 % (RICA occlusion), were placed in the RICA to simulate the development of the stenosis. The stenosis degrees were defined according to the definitions of the North American Symptomatic Carotid Endarterectomy Trial (NASCET) [45] (Eq. 1):

1 $$S = \left( {1 - d_{s} /d_{n} } \right) \times 100 \,\%$$

where $S$ is the stenosis degree, $d_{s}$ is the narrowest ICA diameter and $d_{n}$ is the diameter of normal ICA diameter.

**Fig. 3:**
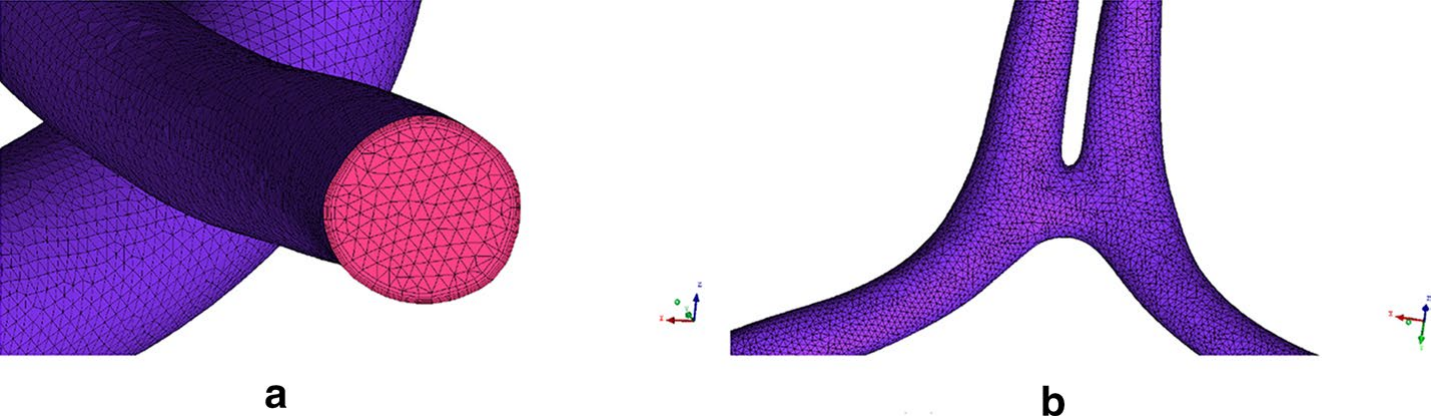
Mesh of **a** vertebral artery (VA) site and **b** ACoA site

The computational grid generation for the flow domain of each case was performed in ICEM software (ANSYS Inc., Canonsburg, PA, USA). The whole flow domain was discretized with an average of 1.37 million mixed tetrahedral and prism cells (Fig. 3).

### Numerical methods and boundary conditions

It has been known that blood behaves as a non-Newtonian fluid, particularly at low shear rates (<100 s^−1^) [46]. In this study, blood was mathematically modelled as a viscous incompressible non-Newtonian fluid by using Carreau model [47]. The Carreau model assumes that viscosity varies according to the following law (Eq. 2):

2 $$\mu = \mu_{\infty } + \left( {\mu_{0} - \mu_{\infty } } \right)[1 + \left( {\lambda \dot{\gamma }} \right)^{2} ]^{(n - 1)/2}$$

where $\lambda = 3.313\,{\text{s}}$, $n = 0.3568$, $\mu_{0} = 0.056\;{\text{Pa}}\,{\text{s}}$, $\mu_{\infty } = 0.00345\;{\text{Pa}}\,{\text{s}}$ and $\dot{\gamma }$ is the shear rate.

For simplicity and to reduce the cost of computations, the elastic properties of the arterial wall were neglected in this study.

Incompressible N-S equations were used as the governing equations (Eqs. 3, 4):

3 $$\rho \left( {\frac{\partial V}{\partial t} + V \cdot \nabla V} \right) = - \nabla p + \mu \nabla^{2} V$$

4 $$\nabla \cdot V = 0$$

In the physiological conditions, cerebral perfusion pressure (CPP) is one of the determining factors that regulate the cerebral blood flow (CBF). CPP is defined as [48]:

5 $$CPP = MAP - ICP$$

where MAP is the mean arterial pressure and ICP is the intracranial pressure. The normal range of the CPP and ICP is 60–100 and 0–15 mmHg, respectively [49].

In this study, we used the CPP as the inlet pressure. To determine the CPP, MAP was calculated by applying the Eq. (6) [50]:

6 $$MAP \cong DP + 0.01 \times { \exp }\,(4.14 - 40.74/HR)\,(SP - DP)$$

where DP is the diastolic pressure, SP is the systolic pressure and HR is the heart rate. Here we take DP = 80 mmHg, SP = 120 mmHg and HR = 80 beats min^−1^, all the values are within the normal range of healthy adults. The calculated MAP is 95 mmHg, the ICP was assumed to be 3 mmHg, and then the CPP could be calculated by using Eq. (5). Thus the CPP = 92 mmHg was determined as the inlet pressure in the current study.

Due to the limitations of the clinical methods, there is a lack of clinical reference values of the pressure at the efferent arteries of the CoW. The clinical measurements indicated that the total CBF in the human brain is 12.5 ml s^−1^ [51]. According to the average value of blood distribution of efferent arteries [52–54], we determined that the blood flow distribution in numerical model is 22 % for ACA, 45 % for MCA and 33 % for PCA. Other than CPP, the CBF is also governed by the cerebrovascular resistance (CVR) [55], which is determined by the distal small arteries, arterioles and capillaries:

7 $$CBF = CPP/CVR$$

Based on the Eq. (7), the lumped CVR values of the ACAs, MCAs and PCAs regions are 0.56 mmHg/ml/min, 0.27 mmHg/ml/min and 0.375 mmHg/ml/min. All the values are within the physiological ranges [56–58]. By using the dimensional analysis method, the lumped CVR values can be interpreted as constant pressure under steady flow conditions. Therefore, the outlet pressures can be calculated based upon the lumped resistance, which are 46 mmHg, 56 mmHg and 43.5 mmHg for each one of ACAs, MCAs and PCAs respectively.

On all the walls and interfaces, a non-slip boundary condition was specified. Due to the relatively small arterial diameter as well as the low flow speed, the Reynolds number is far less than 2100. Thus, the flow condition is assumed as laminar flow [36].

## Results

### Cerebral blood supply

**Fig. 4:**
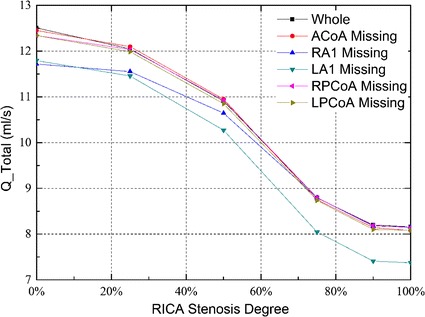
Total flow rates for the six CoW configurations with an increasing stenosis of RICA

Figure 4 shows the changes of the total flow rates of all the cases studied under different degrees of RICA stenosis. The configuration of the missing LA1 presented the highest reduction in the total flow rates for all degrees of RICA stenosis. The specific total flow rates are listed in Tables 1, 2 gives the percentage change of the total flow rates. The total flow rate of the complete CoW with no stenosis in the RICA was set as the reference value for comparing with other cases.

**Table 1:** Specific total flow rates under different RICA stenosis degrees

Stenosis degree (%)	Total flow rates (ml/s)					
	Complete	ACoA missing	RPCoA missing	LPCoA missing	RA1 missing	LA1 missing
0	12.51	12.45	12.34	12.34	11.72	11.79
25	12.04	12.10	12.03	11.99	11.55	11.46
50	10.91	10.95	10.93	10.85	10.64	10.27
75	8.79	8.75	8.80	8.74	8.79	8.05
90	8.20	8.14	8.19	8.10	8.18	7.41
100	8.16	8.07	8.14	8.08	8.16	7.37

**Table 2:** Percentage change of total flow rates under different RICA stenosis degrees

Stenosis degree (%)	Percentage change of total flow rates (%)					
	Complete	ACoA missing	RPCoA missing	LPCoA missing	RA1 missing	LA1 missing
0	0.00	−0.48	−1.36	−1.36	−6.31	−5.76
25	−3.76	−3.28	−3.84	−4.16	−7.67	−8.39
50	−12.79	−12.47	−12.63	−13.27	−14.95	−17.91
75	−29.74	−30.06	−29.66	−30.14	−29.74	−35.65
90	−34.45	−34.93	−34.53	−35.25	−34.61	−40.77
100	−34.77	−35.49	−34.93	−35.41	−34.77	−41.09

**Fig. 5:**
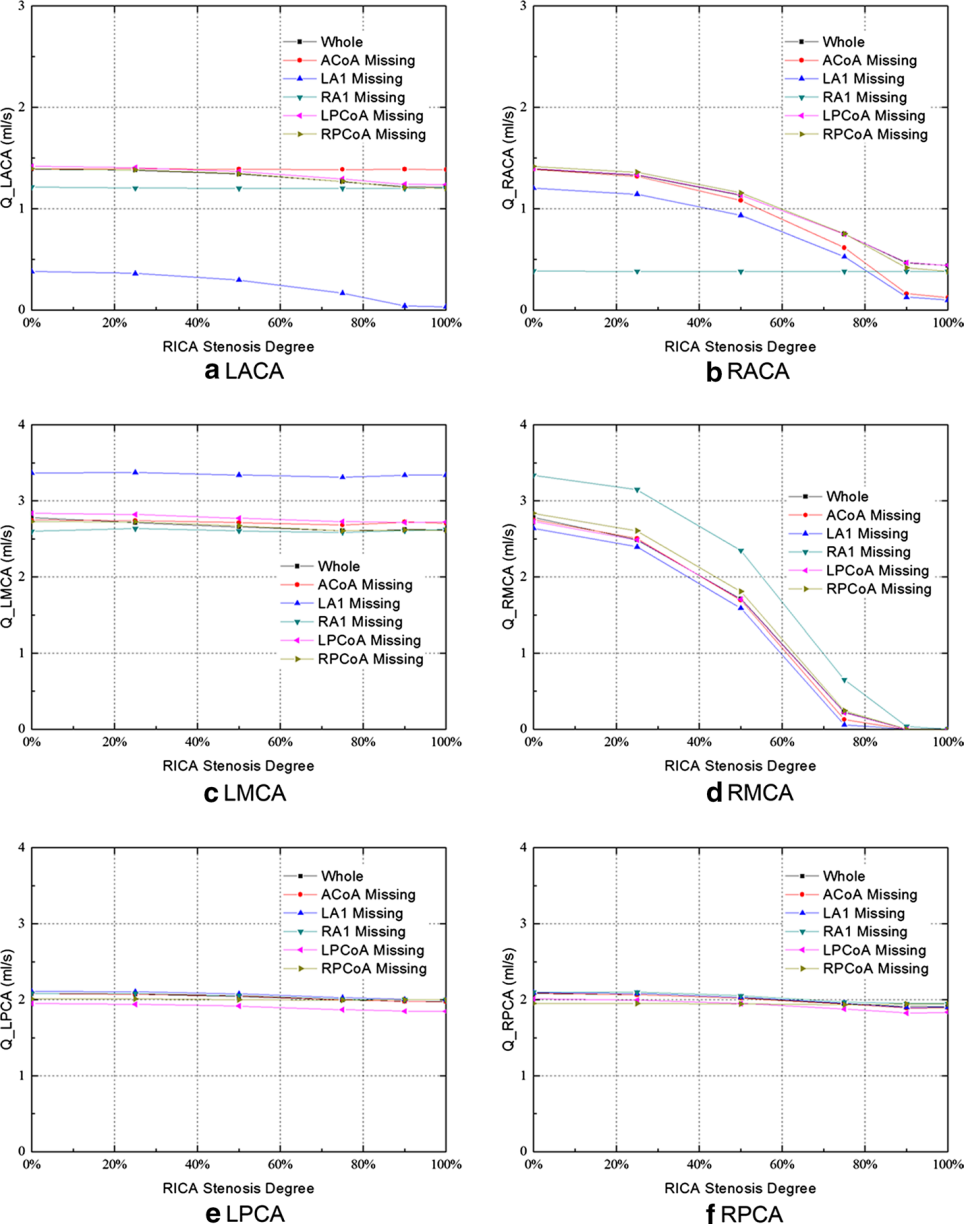
Efferent flow rates for the six CoW configurations with the increasing stenosis of RICA

Figure 5 shows the changes of flow rates in the efferent arteries of all the studied configurations. Except for the absence of RA1, a reduction of the blood supply in the efferent arteries at the affected side was observed in all the cases when the degree of stenosis in the RICA increased. On the contralateral side, the blood flows in MCA and PCA were hardly related to the anatomical configurations, and were almost not affected by the stenosis in the RICA.

In the configuration of RA1 absence, the flow rate in the LACA and RACA was 13 and 73 % lower than that in the normal configuration without RICA stenosis, respectively. The degree of RICA stenosis, however, did not affect the blood supply in bilateral ACAs under this configuration. Moreover, compared with the other studied configurations, an increase of blood flow in the RMCA was observed.

In the configuration of LA1 absence, the flows in the LACA, RACA, and RMCA reached the minimum level, and the blood supply decreased when the degree of stenosis in RICA increased. In particular, the flow rate in the LACA was 0.38 ml/s under no RICA stenosis and was 0.03 ml/s under the occlusion of RICA. The value was 73 and 98 % lower than that in the normal CoW configuration, respectively. Meanwhile, a higher blood flow in the LMCA was observed under this configuration.

In the posterior circulation, the blood supply was hardly influenced by the stenosis in the RICA and the anatomical variations of the CoW. In the LPCA and RPCA, the lowest blood flow occurred when the RICA occlusion coexisted with the absence of the LPCoA. The blood flows were 7 % lower than that in the normal configuration for the LPCA, and 4 % lower for RPCA.

### Flow of the communicating arteries

**Fig. 6:**
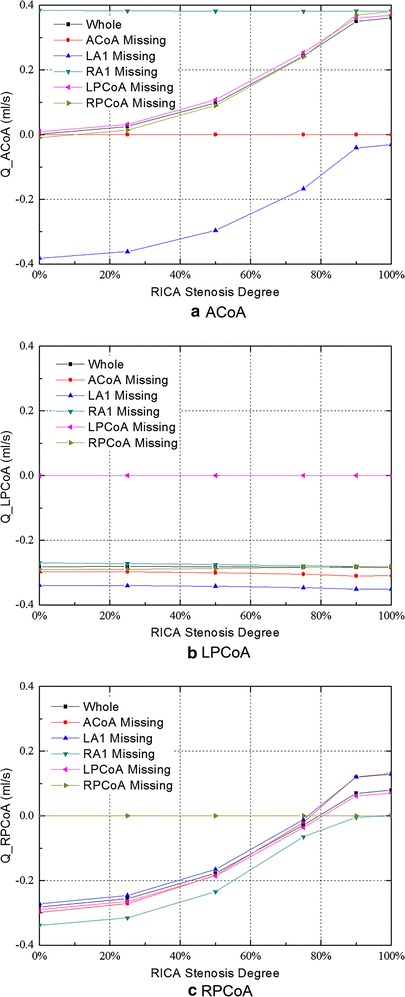
Flow in the communicating arteries for all studied configurations with the increasing stenosis of RICA

Figure 6 shows the flow of the communicating arteries. Flow towards the right in the ACoA and towards the anterior in the PCoA was defined as the positive flow.

In the complete CoW without RICA stenosis, the efferent blood supply was essentially split symmetrically between the left and right, and there is no flow through the ACoA.

In most of the cases, a compensational flow across the ACoA was observed as stenosis appeared in the RICA, and the collateral flow rates in the ACoA increased with an increasing degree of stenosis. The collateral flow travelled from left to right in order to compensate for the blood supply to the affected side. However, two exceptions were observed. First, when the contralateral proximal ACA (A1) was missing, a reversed collateral flow in the ACoA was observed (from the right to the left hemisphere). Meanwhile, the compensational flow rate decreased as the degree of RICA stenosis increased (Fig. 6a). Second, in the absence of RA1, the collateral flow in the ACoA reached to its maximum value (0.38 ml/s) in all the cases even without stenosed RICA, and it remained constant as the degree of stenosis in the RICA increased.

In the normal CoW configuration, only a relatively small amount of blood flow towards the posterior circulation was observed through the bilateral PCoAs (Fig. 6b, c).

The flow rate in the LPCoA was not affected by the stenosis in the RICA and was only correlated with the anatomical variations of the CoW. The collateral flow through the LPCoA reached to its maximum value of 0.35 ml/s when LA1 is absent. Unlike the LPCoA, the flow in the RPCoA was correlated with the degree of RICA stenosis. When the degree of RICA stenosis reached to a certain value, the blood flow direction through the RPCoA switched to feeding the anterior circulation area. The turning point corresponding to each pathological condition is different. When LA1 was absent, the turning point corresponded to the minimum stenosis degree of about 77 %; whereas when RA1 was absent, it corresponds to the maximum stenosis degree of about 99 %, as is shown in Fig. 6c.

## Discussion

Cerebral collateral circulation is the key factor for maintaining the cerebral blood supply of the patients with ICA stenosis. In the present study, we investigated the cerebral blood perfusion in the CoW of different configurations coexisting with various severities of stenosis in unilateral ICA. Furthermore, the collateral circulation mechanism of the CoW was clarified based on the blood distribution patterns.

### The steady boundary condition

Comparing the steady flow results and the pulsatile flow ones in the study of flow in the carotid bifurcation, Ku et al. indicated that the velocities and wall shear stress that obtained from pulsatile flow conditions were similar to those from the steady flow conditions [59]. In the analysis of the flow in the CoW, Hillen et al. reported that a steady model can lead to the similar results as the unsteady one [53]. By comparing the unsteady in vitro results with the steady ones, Chen suggested that the steady pressure inputs, showing no significant difference in the average value of flow rate, could be used in the studies on blood flow in the CoW focusing on average values [60]. Kobayashi et al. also mentioned that the flow phenomena that occurred in pulsatile flow are essentially the same as those found in steady flow in the vertebrobasilar arterial system [61]. A more recent in vitro study further indicated that the capability of the steady flow condition to predict the flow distribution in the CoW [62]. As the aim of this paper was to investigate the cerebral blood distributions in the circle of Willis under various pathological and anatomical variations, the authors mainly focused on the averaged blood supplies through efferent arteries. Other hemodynamic parameters were not within the scope of the current study. Therefore, the steady state boundary conditions are reasonable to be used in this study.

### Validation

**Fig. 7:**
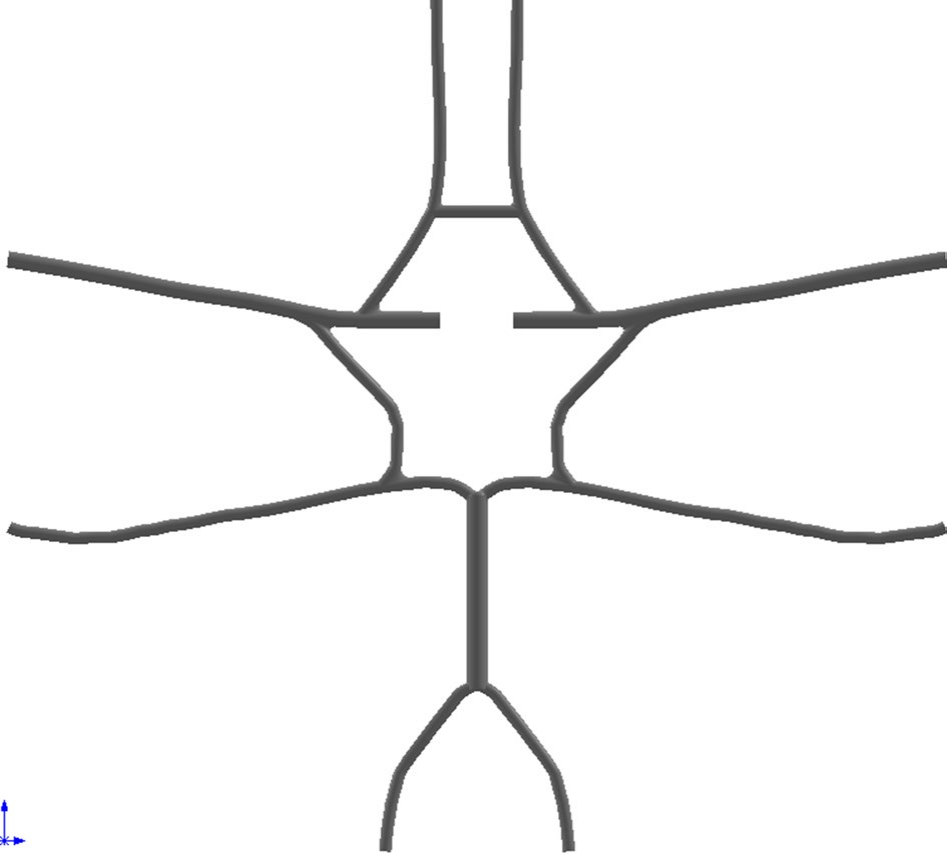
The geometry of the CFD model for validation

In the CFD studies, validation is of the utmost importance if the simulation results are to be trusted. Referring to the guide for CFD validation that published by the American institute of Aeronautics and Astronautic (AIAA) [44] and by Oberkampf et al. [63], a CFD model based on the geometry of the previous in vitro model was developed for the validation. Figure 7 shows the geometry of the CFD model.

The boundary conditions and fluid properties of the previous experimental study [64] were directly adopted to the current CFD simulation. The arterial walls were assumed to be rigid because the deformability was also neglected in the in vitro study. Non-slip boundary condition was applied to all interfaces.

Two cases were studied in the current manuscript to give further validation.

1. Complete circle with no stenosis in RICA
2. Complete circle with 50 % stenosis in RICA.

**Fig. 8:**
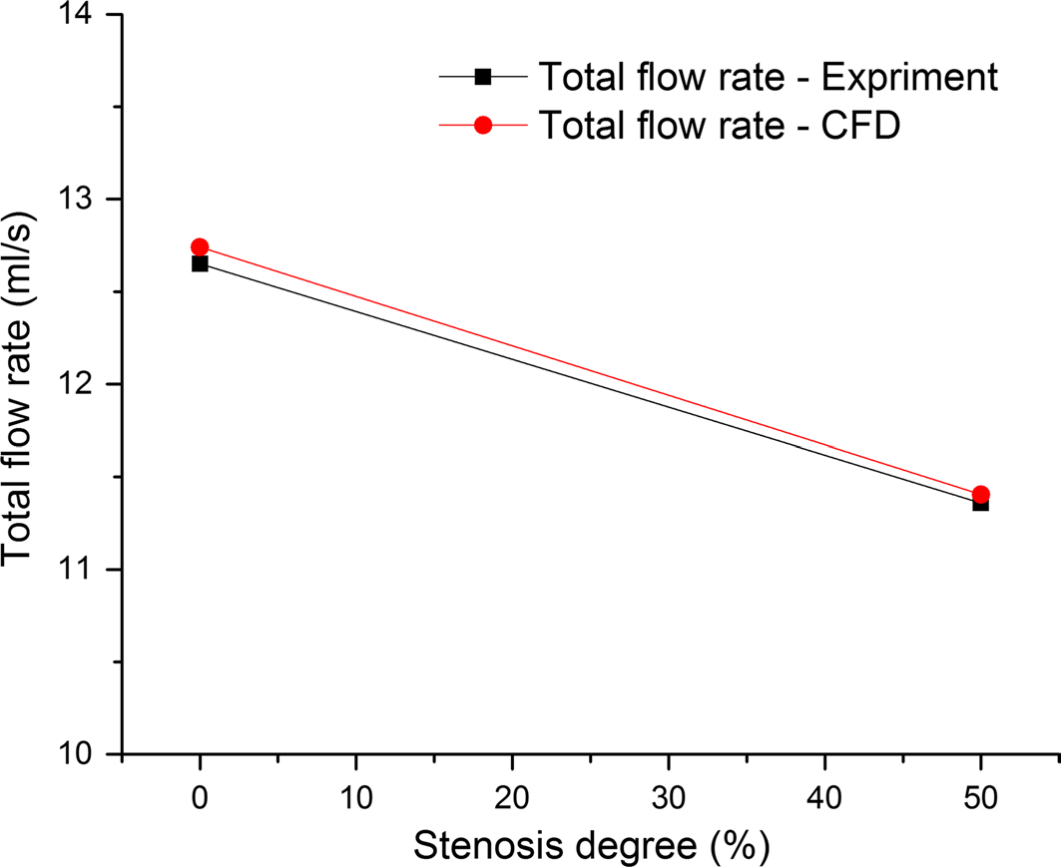
Comparison of the total flow rates between the CFD simulation and the previous experimental study

The flow distribution in the CoW is the main concern of the CFD model. Thus, the total flow rates and efferent flow rates were studied by comparing the results of the simulation and experiment. Figure 8 shows the total flow rates of CFD simulation and in vitro experiment.

**Fig. 9:**
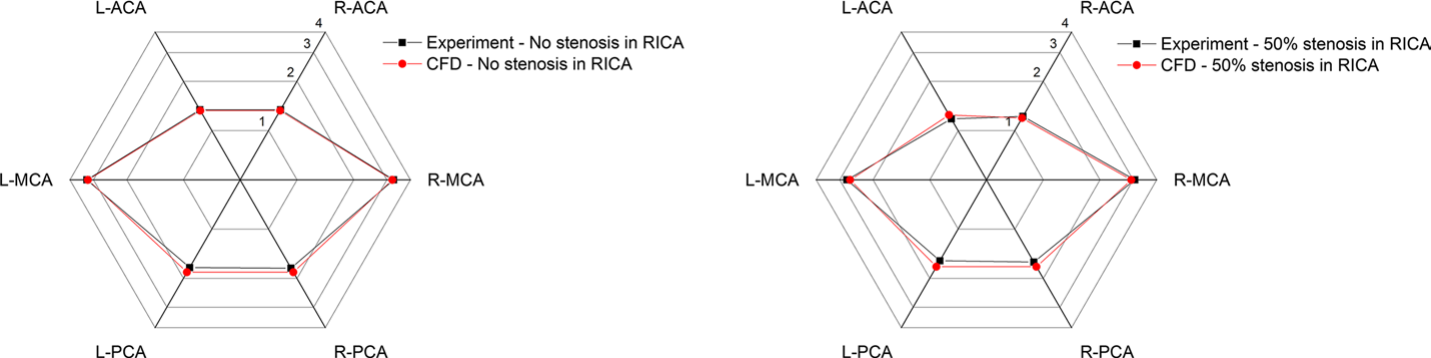
Comparison of the flow rates in the efferent arteries between the CFD simulation and the previous experimental study (ml/s)

Figure 9 shows the flow rates in efferent arteries.

The percentage differences between the results of CFD simulation and that of the in vitro experiment were calculated. The maximum difference was less than 10 %. The detailed information is listed in Table 3.

**Table 3:** Comparison of flow rates between the CFD simulation and the previous in vitro experiment

	Flow rates in the CoW without stenosis			Flow rates in the CoW with 50 % RICA stenosis		
	Experiment (ml/s)	CFD (ml/s)	Percentage difference (%)	Experiment (ml/s)	CFD (ml/s)	Percentage difference (%)
R-ACA	1.34	1.33	−1.17	1.22	1.19	−2.21
R-MCA	3.26	3.21	−1.41	3.06	2.93	−4.18
R-PCA	1.73	1.83	5.85	1.59	1.70	6.67
L-PCA	1.72	1.83	6.56	1.56	1.70	8.78
L-MCA	3.26	3.21	−1.41	2.75	2.64	−3.96
L-ACA	1.34	1.33	−1.17	1.18	1.25	5.59
Total flow rate	12.65	12.74	0.72	11.36	11.41	0.40

During the in vitro experiment and the CFD simulation, the same models were used to give a proper comparison. The results from the simulation match well with that reported in the previous experimental study [64]. And thus, the proposed patient-specific CFD model could be validated in some extent [63].

### Complete model

In the configuration of no anatomical variation in the CoW, the flow was symmetrical in the bilateral efferent arteries, and no blood flowed through the ACoA when there was no stenosis occurred. This indicates that the blood supply to the left and the right hemisphere is relatively independent when there is no anatomical variations existed. Although in contrast with some of the previous studies [62, 65], it is supported by other in vivo [15], in vitro [64, 66] and numerical [36] studies. In the PCoAs, as illustrated in Fig. 6b, c, there is a small amount of flow towards the posterior circulation.

**Fig. 10:**
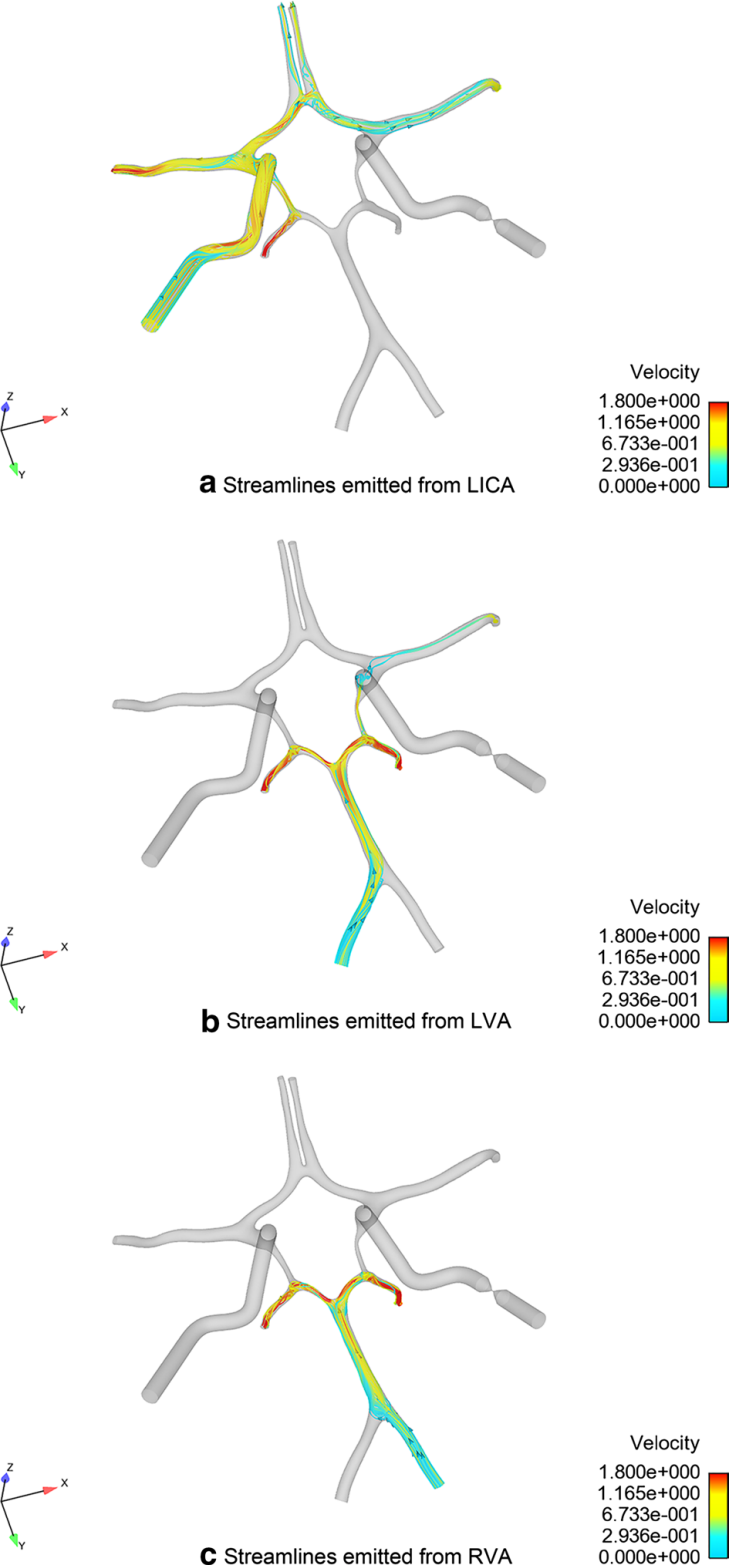
Flow distribution in complete CoW with RICA occlusion

The cross-flow through the ACoA was observed immediately when mild stenosis in unilateral ICA occurred. With the development of unilateral ICA stenosis, flow via the ACoA towards the affected side continuously increased so as to compensate for the lack of perfusion by the stenosed ICA (Fig. 6a). Part of the flow from the ACoA compensated for the blood supply of the ipsilateral ACA and maintained it within a normal range while the stenosis is mild (stenosis degree < 25 %). Nonetheless, if the stenosis degree increased from 0 to 100 %, the blood supply of the ipsilateral ACA would reduce by 68.3 %. The rest of the collateral flow from the ACoA compensated for the blood supply in the ipsilateral MCA (Fig. 10). Compared with the ACAs, the blood supply in the MCAs is more sensitive to the stenosis in the ipsilateral ICA. The perfusions in the MCA ipsilateral to the stenosed ICA are reduced immediately even with mild stenosis. This phenomenon agrees well with the previous numerical [36, 65] and in vitro [64] studies. In the ipsilateral PCoA, flow towards the posterior circulation declined with the growing of stenosis, and the direction was reversed to compensate for the ipsilateral MCA when the stenosis degree was greater than 75 %. In contrast to the affected side, the blood flows in the ACA and MCA at the contralateral side was decreased only by 13 and 6 % when the RICA stenosis increased from 0 to 100 %.

As primarily rely upon the vertebral-basilar system, blood flows in the posterior circulation are hardly influenced by the reduced lumen area of the unilateral ICA. The fluxes through the LPCA and RPCA were reduced by 4.8 and 8.2 %, respectively, when the degree of RICA stenosis increased from 0 to 100 %. The minor drop of perfusion in the PCAs may be due to the redistribution of the blood flow from the anterior circulation and VA so as to compensate for the ipsilateral via the ipsilateral PCoA (Fig. 10c).

The flow rates in the ACAs and MCAs agree well with the results from the previous in vivo and in vitro studies (Table 4). The flow rates in the PCAs, however, appeared slightly larger than those from the in vivo measurements [67, 68]. This could be explained by the absence of the branch arteries of the ICAs and BAs, which may cause the increase of flow in the PCAs [42]. This hypothesis was supported by two in vitro experimental studies [64, 69] published previously.

**Table 4:** Comparison of flow rates in efferent arteries of complete CoW with previous in vivo measurements

Study	Volume flow rate ml/s					
	LACA	RACA	LMCA	RMCA	LPCA	RPCA
Current model	1.39	1.39	2.78	2.78	2.08	2.08
Enzmann et al. [67]^a^	1.25 ± 0.17	1.47 ± 0.18	1.80 ± 0.12	2.12 ± 0.12	0.88 ± 0.07	0.85 ± 0.07
Zhao et al. [68]	1.42 ± 0.43	1.33 ± 0.47	2.5 ± 0.52	2.42 ± 0.45	1.10 ± 0.23	1.05 ± 0.23
Cieslicki et al. [69]	1.54 ± 0.03	1.56 ± 0.03	2.44 ± 0.05	2.41 ± 0.05	1.88 ± 0.04	1.88 ± 0.04

^a^Mean blood flow of efferent arteries

The data presented in the previous in vivo and in vitro studies showed that the current CFD results are within acceptable physiological ranges. Therefore, the results of the current study are capable of providing convincing information about the CBF distribution and revealing the collateral circulation mechanism under such situations.

### Absence of ipsilateral proximal ACA (A1)

Flow in the efferent arteries exhibited very distinctive patterns when the ipsilateral A1 is missing. Except for the ipsilateral MCA, the blood flows in all of the efferent arteries remained constant when the lumen area of the unilateral ICA was reduced. This phenomenon is possible relate to the characteristic of the structural variation. Because only a small amount of blood supply flowed through the PCoAs (Fig. 6b, c), the left and right anterior circulations of the CoW were disconnected when the unilateral A1 was missing. Thus, the blood flows in the efferent arteries on the healthy side was not influenced by the stenosed ICA. The cerebral perfusion in the ACA on the affected side is also solely relied on the blood supply from the healthy side and was not influenced by the development of stenosis in the ipsilateral ICA.

In this configuration, the flow in the ACoA reached to the maximum value among all of the investigated configurations, and it remained constant while the stenosis degree in the unilateral ICA increased.

### Absence of contralateral proximal A1

When the contralateral A1 was absent, the blood flows in the ipsilateral MCA and bilateral ACAs, together with the total flow rate all reached to the minimum value among all the studied cases. This important observation implies that LA1 absence is the most dangerous situation in terms of cerebral blood supply.

In this configuration, the contralateral ACA was totally disconnected from the blood supply of the healthy side and, therefore, relied only on the compensational flow from the affected side via the ACoA. This is the only case in which the flow in the ACoA is reversed, from the right to the left. With the increased degree of stenosis in the unilateral ICA, the compensational flow to the contralateral ACA decreased due to the decreased blood supply from the stenosed ICA. At the same time, the blood supplies in the ipsilateral MCA and ACA also reduced due to a lack of collateral compensation from the healthy side. Flows in the posterior circulation, as discussed before, are not influenced by the stenosis in the ICA in this configuration.

### The function of the communicating arteries

In the absence of ACoA, the collateral channel between the left and right side of the CoW is cut off. Though the flow distributions in this configuration are similar to that in the complete CoW, the ipsilateral ACA lost its collateral feeding source, which may subsequently lead to a greater reduction in the blood supply when the ICA stenosis occurred. Flow in the ACA at the contralateral side remained constant regardless of the severity of stenosis. With the absence of ACoA, the flow towards the posterior circulation via the contralateral PCoA increased (Fig. 6b). Some of the blood supply flowed through the bilateral PCAs and ipsilateral PCoA and then compensated the starving ipsilateral MCA. Because of the newly established collateral pathway, flow in the ipsilateral MCA was not influenced by the absence of the ACoA even though it also lost its collateral blood supply from the ACoA.

In the PCoAs, as illustrate in Fig. 6b, c, there is almost no flow in the PCoAs in the complete CoW. When the stenosis in the unilateral ICA occurred, the ipsilateral PCoA provides an important collateral pathway to feed the anterior circulation. This observation is corroborated by clinical TCD measurement [15]. The contralateral PCoA, however, almost provides no support to the collateral blood supply.

### Limitations of the study

Several limitations of the current study are as follows. First, some vessels of the CoW, including the ophthalmic artery, choroidal arteries, and superior cerebellar arteries, were simplified due to the difficulty of modelling reconstruction. As discussed before, the simplification would influence the flow distribution in the CoW in some extent.

Secondly, the arterial walls were assumed to be rigid. Though has little impact on the spatial flow distribution [40, 70], the negligence of the arterial elasticity constrained the capability of providing accurate wall shear stress (WSS) distribution [71–75] and investigating the propagation patterns [76, 77] of the current model. This limitation can be improved in the future studies that aim to investigate more detailed hemodynamic parameters in cerebral arteries.

Moreover, the cerebral autoregulation (CA) mechanism was not considered in the current study. This simplification may lead to an underestimation of cerebral perfusion as well as an overestimation of the cerebral perfusion impairment [39, 78–81]. The quantitative impact of the CA in different models, however, are varied [82]. Thus, our current model based on the assumption of neglecting the CA is still capable of providing information to analyse the aspects the brain circulation. But the findings should be interpreted with cautions when come to the biological and clinical significance.

## Conclusions

The cerebral perfusions and the collateral mechanism in the CoW of different configurations have been investigated numerically in a patient-specific model. The simulations showed in the complete CoW, ACoA and ipsilateral PCoA functioned as the important collateral channels when stenosis in unilateral ICA occurred. The cross-flow in the ACoA is a sensitive indicator of the morphological change of the ICA. The collateral function of the ipsilateral PCoA will not be fully activated until a severe stenosis in unilateral ICA occurred. The configuration of contralateral A1 absence with severe stenosis in unilateral ICA presents the highest risk of ischemic stroke. The findings of this study would enhance the understanding of the collateral mechanism of the CoW under different anatomical variations, which will eventually lead to therapeutic and diagnostic applications in the future.

## Abbreviations

ACA: anterior cerebral arteries
ACoA: anterior communicating artery
BA: basilar artery
CBF: cerebral blood flow
CoW: circle of Willis
CT: computed tomography
CTA: computed tomography angiography
ICA: Internal carotid arteries
L: left
A1: proximal anterior cerebral artery
MCA: middle cerebral artery
PCA: posterior cerebral arteries
PCoA: posterior communicating artery
R: right
TCD: transcranial doppler
TIA: Transient ischemic attack
VA: vertebral artery
